# Current Progress in CAR-T Cell Therapy for Hematological Malignancies

**DOI:** 10.7150/jca.48976

**Published:** 2021-01-01

**Authors:** Donglei Han, Zenghui Xu, Yuan Zhuang, Zhenlong Ye, Qijun Qian

**Affiliations:** 1 Henan Cell Therapy Group Co. LTD, Zhengzhou, Henan, China.; 2 Shanghai University Mengchao Cancer Hospital, Shanghai, China.; 3 Shanghai Baize Medical Laboratory, Shanghai, China.

**Keywords:** CAR-T cells therapy, hematological malignancies, safety strategies

## Abstract

Immunotherapies, such as monoclonal antibody therapy and checkpoint inhibitor therapy, have shown inspiring clinical effects for the treatment of cancer. Chimeric antigen receptor T (CAR-T) cells therapy was an efficacious therapeutic approach treating hematological malignancies and encouraging results have been achieved. Three kinds of CAR-T cell therapies, Kymriah (tisagenlecleucel), Yescarta (axicabtagene ciloleucel), were approved for clinical application in 2017 and Tecartus (brexucabtagene autoleucel) was approved in 2020. Despite some progress have been made in treating multiple hematologic tumors, threats still remain for the application of CAR-T cell therapy considering its toxicities and gaps in knowledge. To further comprehend present research status and trends, the review concentrates on CAR-T technologies, applications, adverse effects and safety measures about CAR-T cell therapy in hematological neoplasms. We believe that CAR-T cell therapy will exhibit superior safety and efficacy in the future and have potential to be a mainstream therapeutic choice for the elimination of hematologic tumor.

## Introduction

Treating cancer with CAR-T cells has made significant achievement and has gradually become a crucial approach in healing hematological malignancies 1. Kymriah and Yescarta were the first batch of CAR T-cell drugs to be recommended by FDA in the United States for treating leukemia and lymphoma in August 2017 and October 2017 2. FDA approved another CAR T-cell drug named Tecartus for treatment of adult patients diagnosed with mantle cell lymphoma (MCL) in July 2020. CAR-T cell therapy as an innovative treatment method has potential to change the clinical outcome for many patients, but challenges still exist to be settled before the therapy turns into a dominated therapeutic choice considering its security and effectiveness 3. The development of CAR-T cell treatment is impeded by serious adverse reactions, such as the release of cytokine, neurotoxicity, tumor lysis syndrome, off-tumor target toxicity, as well as labor-intensity and high cost. On the other hand, the occurrence of recidivation is also a primary challenge.

Despite many issues, CAR-T cells therapy is indisputably a promising tool for the future adoptive cancer immunotherapy and increasing clinical trials for blood cancer have been registered. Unlike general T cells, CAR-T cells are not restricted by major histocompatibility complex (MHC) when they recognize tumor-associated antigens (TAAs). Encouragingly, many methods of reducing cytotoxicity and increasing CAR-T cells' curative effect have been developed and have made significant progress through regulating the CAR-T cells cytoactivity 4. In order to ensure long-term benefits, future studies may need to seek targets with enhanced safety and efficacy or incorporate novel CAR constructs. There's numerous evidence showing that combination therapies were also decent choices to enhance therapeutic efficacy. CAR-T cells therapy combined with checkpoint blocking antibodies or small molecule inhibitors have shown inspiring results than single-drug approach.

## The mechanisms and development of CAR-T cell therapy

The technique of CAR-T cells that expressing antigen receptor was first designed by Eshhar's team 5. Complete remission treated by CAR-T cells was first covered by Stephan's group for curing patients with acute lymphoblastic leukemia 6. In **Table 1**, we listed the main progresses of Car-T cell therapy.

### The molecular mechanisms of CAR-T cell therapy

Over the years, with the development and progress of genetic engineering, immunology and other related disciplines, CAR-T cell therapy become a new and promising strategy for treatment of blood cancer. CAR-T cell consists of two parts, T cell separated from peripheral blood and CAR designed through genetic engineering. CAR is expressed in the surface of T cell after cDNA integrated in the target cell genome. The genes of CAR are generally transduced into T cells by lentivirus, retrovirus and other ways 1. The deficiency of CAR-T cells is that there is no regulation of the CAR gene expression in consideration of the recognition is independent of MHC manner. That means the immune response of CAR-T cells may out of control and cause some side effects.

The main structure of CAR is composed of a tumor-antigen receptor and signal transduction domain 7. Tumor-antigen receptor recognizes specific TAAs including protein, glycoprotein and other elements while signal transduction domain principally enhances T-cell proliferation and differentiation. Different generations of CAR structure are charactered with diverse intracellular signal transduction domain 8.

**Figure 1:**
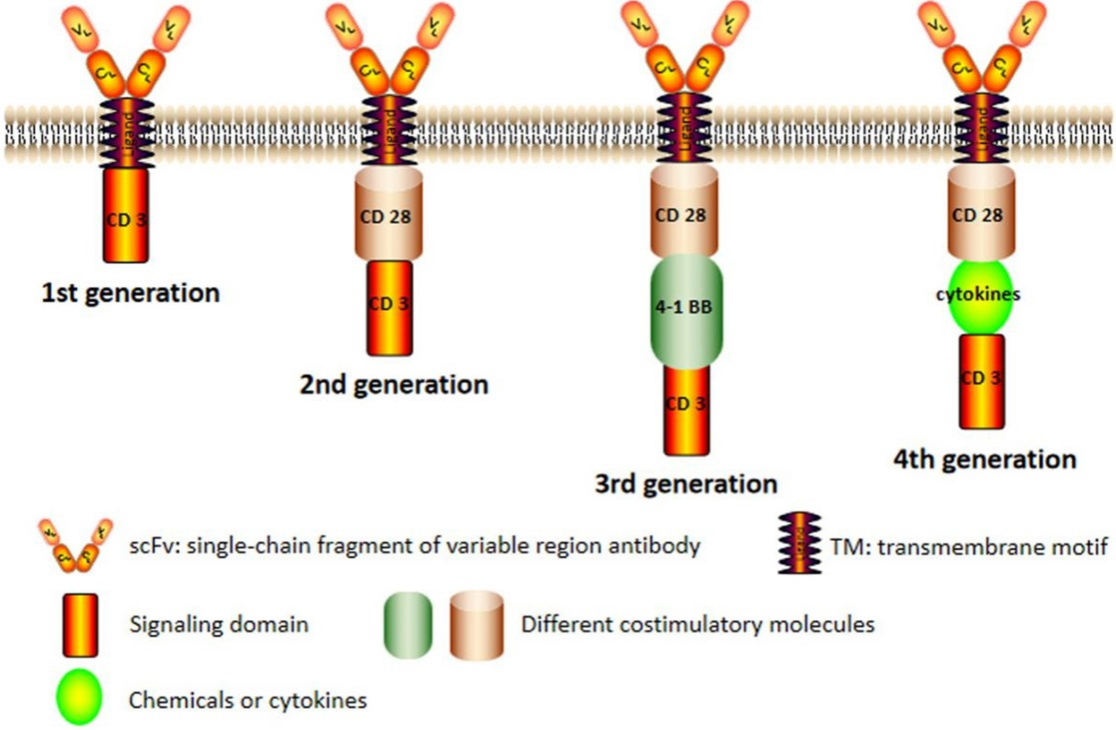
** The chimeric antigen receptor (CAR) structure of four generations.** The conventional structure of CAR (second generation) including a single-chain fragment of variable region antibody, a transmembrane domain, a costimulatory molecule and an intracellular signal domain. The CAR of first generation was basically eliminated because its weak effect. In the third generation, there were more than one costimulatory molecules and there added some chemicals or cytokines in the fourth generation.

CAR structures of four generations are shown in **Figure 1.** The original framework of CAR merely has an intracellular CD3ζ signaling module in which signal transmission is oversimplified and impotent 9. Though the CARs of the first generation can specifically recognize tumor antigens and elevate anti-tumor activity of T cells, the therapeutic effect is not satisfactory *in vivo* for its decreased proliferation ability 10. The second generation of CAR, which integrates a costimulatory domain such as CD28 or 4-1BB with CD3ζ molecule, has exhibited remarkable improvements in cell multiplication and senescence 11.

The third generation aggrandizes another costimulatory molecules in signal transduction domain based on the second generation. The CAR of the third generation contains two different costimulatory signals simultaneously, such as CD28 and CD137. It still exists the fourth generation of CAR expressed on T cell surface, T cells redirected for universal cytokine killing (also called TRUCK) which can secrete important chemicals or cytokines in tumor tissues, improving the tumor cytotoxicity by overcoming the immunosuppressive network in the tumor microenvironment. Despite some issues about safety and efficacy, the CAR-T immunotherapy is indisputably a promising way for treating hematological malignancies in the future.

### The cellular mechanisms of CAR-T cell therapy

Mutated cells and cancer cells can be recognized and eliminated by immune cells. T cells depend on TCR structure and the presentation of MHC to identify TAAs that expressed in cancer cells, while CAR-T cells only depend on engineered CAR structure. CAR-T cells have both antigen binding specificity of CARs and cytotoxicity of T cells. CD19 is the most commonly applied TAA in the treatment of hematological neoplasms. CD19 CAR-T cells are designed and expanded *in vitro*, when injected into the body they attack all CD19-positive cells including normal CD19-positive cells 6.

Once binding specific TAAs, CAR-T cells initiate activation through phosphorylation and proliferation to a large number. Anticancer response is mainly via cytotoxicity and cytokine secretion. CD8-positive CAR-T cells play an essential character in destroying tumor cells. CD4-positive CAR-T cells execute assisting role that can strengthen anti-tumor immune reaction. CAR-T cells perform cytotoxicity by secreting granzyme and perforin that can damage tumor cells. The other way of cytotoxicity is stimulating apoptosis induced by activation of apoptotic signaling pathways within cancer cells. Cytokines released by CAR-T cells enhance tumor clearance through activating multiple immune cells and generating synergy effects 12.

## The applications of CAR-T cell therapy in hematological neoplasms

By now, the CAR-T cell therapy is widely applied to various oncotherapies, especially in hematological malignancies 13. In this section, the latest study and application of the CAR-T cell therapy in leukemia, lymphoma and multiple myeloma were introduced (**Table 2**).

### CAR-T cell therapy applied in acute lymphoblastic leukemia

CAR-T cytotherapy have demonstrated markedly efficacious in curing acute lymphoblastic leukemia (ALL), especially suitable for fatal relapsed or refractory B-ALL. CD19, a crucial molecular marker of B cells, is almost an ideal target in treating B-ALL for its higher expression in the surface of tumor cells. The results are inspiring and satisfactory that the clinical therapeutic effect is accord with expectation. Kymriah developed by Novartis is a CD19-specific CAR-T cells drug that has been approved by the FDA.

Some patients gradually became not sensitive to CD19-specific CAR-T cells because of “antigen escape” of cancer cells. For this problem, additional molecule targets on tumor cells appearance are imperative to be discovered instead of CD19 14. Fortunately, researchers found CD20 and the results showed it can be a potential target 15. CD22 molecule can possibly be a choice for some relapsed patients treated with CD19-specific CAR-T cells and two different anti-CD22 agents are currently under clinical investigation 16. CD123 is another desirable target and preclinical studies have showed promising effects in mice models 17.

### CAR-T cell therapy applied in chronic lymphoblastic leukemia

Chronic lymphoblastic leukemia (CLL) can generate inchoate immune deficiency and lead to more complicated clinical symptoms than ALL. The conventional treatment is allogeneic stem-cell transplantation cooperated with chemotherapy or radiotherapy. In recent years, researchers begin to explore the application of CAR-T cell therapy for relapsed and risky CLL, in which CD19 CAR-T cells were mostly used 18. Some clinic trials showed decent curative effect though with limited ability of cell expansion and proliferative response for the immune deficiency of CLL patients 19. In one trial, a patient demonstrated a CR after injected with anti-CD19 molecule CAR-T cells 20. A series of chronic toxic effect, such as B-cell depletion, tumor lysis syndrome, always accompanied with CAR-T cells treatment. To address this issue, plenty of methods are explored by the researchers. Interleukin are used to facilitate anti-tumor effects and suicide genes are useful for controlling acute toxic effects 21. To comprehend explicitly, further exploration is needed and lots of relevant clinical test are in progress.

### CAR-T cell therapy applied in lymphoma

The most traditional therapy for lymphoma including non-Hodgkin lymphoma (NHL), anaplastic large cell lymphoma (ALCL), Hodgkin lymphoma (HL) was chemotherapy regimens and monoclonal antibody. The agents above showed clinical success to a certain extent, still some patients experienced disease deterioration after these therapies 22. A neoteric and valid therapy for lymphoma is of necessity and CAR-T cytotherapy is the ultramodern advanced immunotherapy that used for intractable B-cell lymphoma or patients with poor prognosis after primary therapies 23.

Studies investigated the efficiency of CD19 CAR-T cells for patients who suffered from lymphoma and showed promising consequence that 75% of patients achieved PR 24. Besides CD19, CD20 or CD30 are also essential in treating lymphoma by using CAR-T cells. CD20 is presented in overwhelming majority of B-cell lymphomas and CD20 CAR-T cells have produced sensational clinical utilities in multitudinous NHL treatments 25. Anti-κ/λ CAR-T cells are also a feasible strategy for the treatment of B-cell lymphoma in consideration of the κ or λ light immunoglobulin chain expressed in some mature malignant B cells 26.

### CAR-T cell therapy applied in multiple myeloma

Multiple myeloma (MM) is a refractory hematologic tumor featured by the integration of plasmocytes in medulla ossium that can lead to anemia, immunosuppression, hypercalcemia, bone lesions and renal failure 27. The disease is almost incurable using chemotherapy or autologous HSCT because cellular heterogeneity and molecular deformities of myeloma. Anti-CD19 CAR-T cytotherapy was incapable of demonstrating good efficiency to eliminate myeloma cells though it performs excellent in treating ALL or other hematologic malignancy in consideration of lower expression of CD19 in myeloma cells' surface 28.

The molecule of CD269 (BCMA) which expressed mostly in mature B cells or plasma cells has been studied and is a promising target in MM 29. Another molecule involved is CD138 which is expressed on malignant plasma cells. Clinical trial showed that CD138 CAR-T cells entered the bone marrow after infusion and had potential anti-tumor immunity without intolerable toxicity. More specific targets presented on tumor cells should be explored and more clinical trials should be executed after the fundamental research. Technique breakthrough in MM immunotherapy is urgently needed.

### The interactions of CAR-T cell therapy with other therapeutics

For ages, the basic treatments of cancer have included surgery, chemotherapy, radiotherapy and immunotherapy. For hematologic malignancies, chemotherapy is the prior treatment and HSCT is usually the conventional and exclusively treatment. In recent years, with the development of bioengineering techniques, some newly approved immunotherapies have been developed and showed potential effectiveness in hematologic malignancies. As a new mode therapy for malignant tumor, CAR-T cell therapy has brought incredible promise for hematological cancers. It has greatly improved the possibility of curing malignant tumors especially in relapsed and refractory conditions and is deemed as one of the highly desirable therapeutic modalities. Combination therapies are playing an increasingly important character to enhance therapeutic efficacy and a lot of researches about small molecule combination therapy are underway. A number of potential synergies may arise through combinatorial approach.

The engineering of CAR-T combined with checkpoint blocking antibody or small molecule inhibitors, may present extraordinary therapeutic effect and increase the curative ratio of hematological malignancies. Investigators devote to screening small-molecules to incorporate into CAR-T manufacturing process, attempting to improve the therapeutic properties of the product. In the future, the radical cure for hematologic malignancy not only needs CAR-T cytotherapy but also requires multiple approaches synergism together.

## Side effects of CAR-T cell therapy

**Figure 2:**
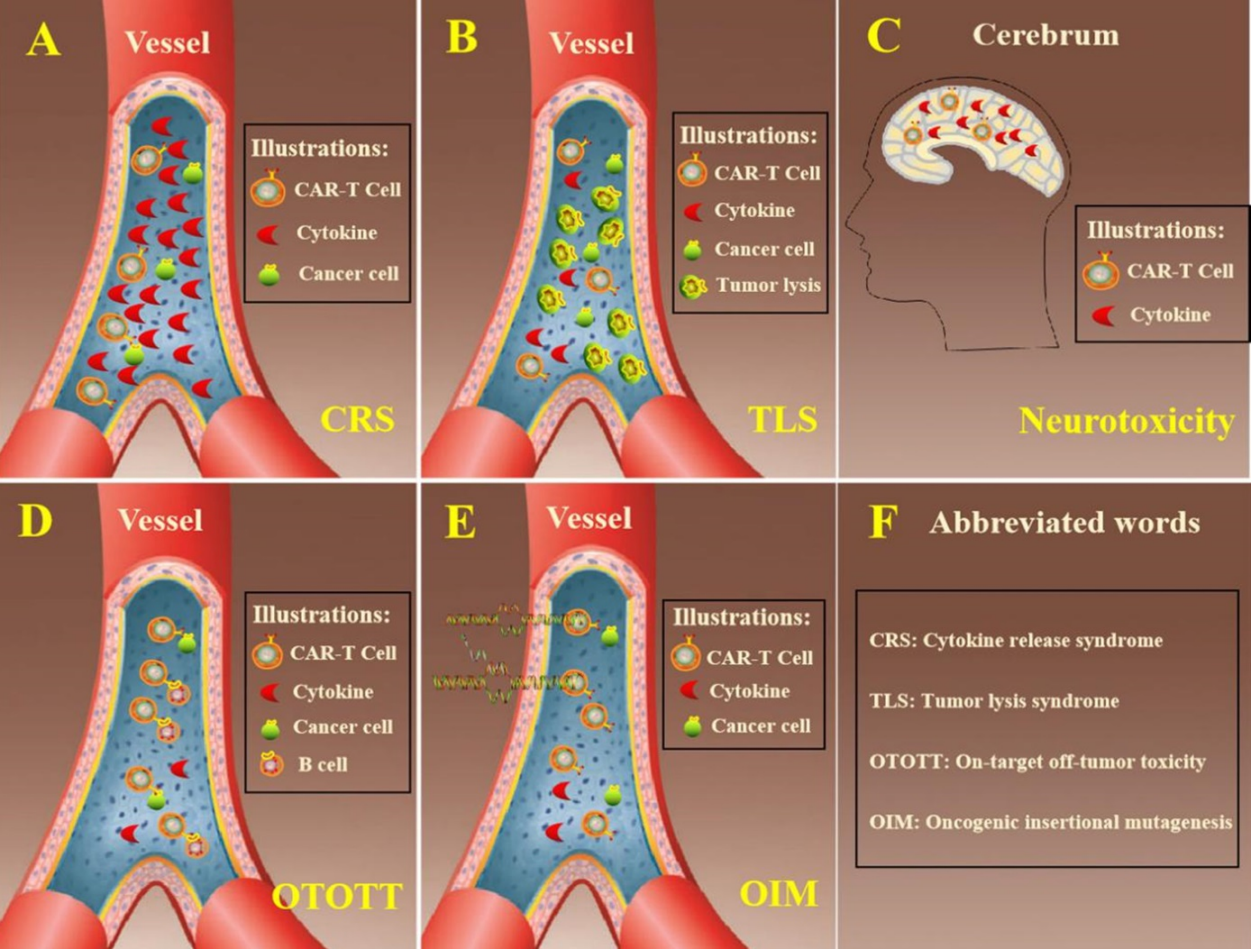
** Summarized adverse effects of CAR-T cell therapy.** A. In the process of eliminating cancer cells, CAR-T cells released excessive cytokine that exceeded circulatory system load. B. Cancer cells were lysed within a short time and too much intracellular substances were released in hematologic system. C. The cytokines secreted by CAR-T cells could penetrate the blood-brain barrier and leaded to neurotoxicity. D. CAR-T cells eliminated tumor cells through recognizing specific antigen in the surface of tumor cells. On the other hand, it also destroyed normal cells at the same time. E. The genes needed always putted into T cells by virus vectors and it is risky that cells might occurred oncogenic insertion mutations. F. The explanation of abbreviated words in the figure.

CAR-T cytotherapy has acquired superduper therapeutic effects in treatment of hematological malignancies, for instance ALL, CLL and NHL. Toxic effects are often accompanied with regular curative effect when patients received CAR-T cells. Further development is impeded with lots of adverse effects that are shown in **Figure 2**, exempli gratia the release of cytokine, neurotoxicity, tumor lysis syndrome and other toxicities. Besides, the general therapeutic effect of CAR-T cytotherapy is reduced by the high rate of relapse and the prevalent clinical application is hindered by the complexity of production and high cost.

### Cytokine release syndrome

The cytokine release syndrome (CRS) is one of the most frequent adverse reactions of CAR-T cell immunotherapy 30. The structure of costimulatory molecules within the CAR-T cells can stimulate cell activation, proliferation, increase cell killing capacity when the receptor molecules bound to the corresponding antigen target and release excessive cytokines. Plethoric cytokines were released to circulatory system, which can bring diverse clinical symptoms, including pyrexia, racing heart, low blood pressure, latently leading to acute respiratory distress syndrome or death 31. As reported, a certain degree of CRS is desired in clinical response because cytokines represent the CAR-T cells were activated and functioning after patients receiving moderate amount of CAR-T cells 32. To better guard patients, it is useful to grade CRS which can guide the dosage management of CAR-T cells. The severity of CRS is in connection with CAR-T cell dosage which has unclear association with clinical outcomes.

### Tumor lysis syndrome

Tumor lysis syndrome (TLS) is also a common toxicity when curing hematological malignancies with CAR-T cells. After the mass destruction of cancer cells, the quick release of cellular contents exceeded the function of liver metabolism and kidney secretion. The accumulation of metabolites leads to a variety of metabolic abnormalities and breaks the homeostasis. The symptoms of TLS had some similarities or overlap with CRS in some respects and can also lead to life-threatening severe arrhythmia 33.

### Neurotoxicity

Neurotoxicity is a common trait during the therapeutic process of CAR-T cytotherapy and it is customarily associated with CRS. When patients were infused with CAR-T cells, the genetically modified T cells were not only detected in the sanguis, but also in the cerebrospinal fluid and cytokines were found in the cerebrum. Cerebral CRS caused by high level of cytokines in cerebrum potentially leads to neurotoxicity, which was generally characterised with delirium, expressive aphasia, seizures and syncope 34. In clinical practice, corticosteroid was one of the choices for the management of neurotoxicity considering its capacity of penetrating blood-brain barrier while lots of monoclonal antibodies were incapable 35.

### Off-tumor target toxicity

The on-target off-tumor toxicity (OTOTT) is an unavoidable side effect because tumor-specific antigens are not only found in tumor cells 36. Tumor cells can be attacked by CAR-T cells which possessed anti-tumor activity, while at the meantime it killed some normal cells 37. CD19 was an ideal target in the surface of tumor cells of most patients with hematologic malignancies while CD19 was also an essential biomarker of B cell lineage. CAR-T cells have achieved superior clinical performance in hematologic neoplasms but was accompanied with B-cell aplasia, which was a major dilemma for extensive adhibition of CAR-T cytotherapy in hematologic malignancies 38.

### Oncogenic insertional mutagenesis

Modified CAR-T cells are conventional transfected with virus vectors that carry specific antigen receptor gene. There is a potential risk of oncogenic insertion mutations (OIM) when CAR-T cells containing specific gene are infused into patients. Though it never occurs that cells immortality was induced by virus vector, it is worth noting that clinical applications of CAR-T cytotherapy, especially in longstanding surveillance, may result in genotoxicity 39.

## Strategies to surmount adverse reaction

The CAR-T cytotherapy has acquired great achievements, but the therapy also comes with many adverse effects. The traditional ways such as administrated corticosteroids or corresponding cytokine inhibitors played a certain function, but very limited. New methods are demanding. Fortunately, researchers have proposed a variety of safety strategies to reduce these side reactions, such as the suicide genes, synthetic Notch receptors and bispecific T cell engagers. Many clinical trials have begun to apply these methods to alleviate cytotoxicity of CAR-T cells.

### On-off via suicide gene

Herpes simplex virus thymidine kinase (HSV-tk) has been widely applied in the treatment of various malignant tumors combined with ganciclovir (GCV) 40. HSV-tk can phosphorylate the GCV, a nucleoside analogue, and form toxic GCV-triphosphate compound which is a substrate can binds to DNA instead of triphosphate. DNA replication was inhibited when incorporated with GCV-triphosphate and subsequently lead to cell death. CAR-T cells designed with HSV-tk suicide gene also displayed predicting results in treating acute myeloid leukemia, that CAR-T cells showed antitumor efficacy and eliminated tumor cells effectively with the existence of GCV 41.

The inducible caspase 9 (iCasp9) safety switch is another frequently used suicide gene. In some clinical trials, iCasp9 suicide gene and its inducer was exploited to eliminate the activated T cells on the basis of CD19 CAR and obtained superior and promising consequence 42.

### Switch via intracellular micromolecule linkage

Intracellular micromolecule linkage was a new strategy that controlled the activities of CAR-T cells. It consists of two mutually independent parts in intracellular space, one part possessing costimulatory domains and the other containing downstream signaling molecules 43. The integrated two parts triggered by priming small molecule is the precondition of therapeutic activity of CAR-T cells. The recognition of specific antigens and the activation of micromolecules led to antineoplastic effect of engineered T cells, which could not be activated by small molecules or antigens alone. The vitality of CAR-T cells with on-switch gene could be regulated by appropriate dosage at proper time in a titrable manner through the small molecule, and then alleviated toxicity. Researchers proposed “transient CAR-T cell” strategy by controlling the amount of small molecules. The two separated parts dimerized with the existance of small molecules and switched CAR-T cells from dormant to activated state. The reaction intensity of modified CAR-T cells was dependent on the quantity of small molecules.

### Combinatorial target-antigen recognition

Combined target-antigen identification means the intracellular structure of conventional CAR is divided into two reciprocal parts which are integrated with different extracellular scFv, respectively. The novel CAR-T cells could recognize two different antigens in the surface of tumor cells. One antigen was recognized by CD3ζ-linked scFv, and another antigen was identified by costimulatory linked scFv. It was a hopeful approach to promote the security of CAR-T cytotherapy by mitigating “off-tumor target” toxicity through assembled target-antigen identification. On the other hand, the recognition of two different antigens reduced antigen escape of tumor cells and enhanced anti-tumor accuracy.

### Synthetic notch receptors

Synthetic Notch (synNotch) receptors were a novel kind of modular receptors that was designed on the basis of Wild-type Notch protein 44. It has been applied in CAR-T cells to improve safety recently. SynNotch receptor recognized one specific tumor antigen and then transcriptional activation domains were released, promoting the expression of a CAR structure. Subsequently, anti-tumor activity was activated after CAR-T cells were targeted with another tumor antigen. The researches indicated that CAR-T cells equipped with synNotch receptors eliminated tumor cells with high efficiency and injured normal tissue cells slightly 45.

### Inhibitory chimeric antigen receptor

Inhibitory chimeric antigen receptor suppressed the immune response through attenuating T cells activity 46. Application of inhibitory receptors, for instance PD-1 and CTLA-4, to CAR-T cells modification is also a prospective safety tactics. Studies showed that the activity of CAR-T cells was restricted in proliferation, cytokine secretion and cytotoxicity when added with PD-1 or CTLA-4 and the activity recovered after removing inhibitory receptors. Therefore, inhibitory chimeric antigen receptor can serve as a safety switch to control response intensity and precaution against off-tumor target adverse effects in a reversible manner.

### Bispecific T cell engager

Bispecific T cell engager is a skill that combines CAR-T cells with specific antigen in tumor cells through bispecific small molecules that was always constituted of a fluorescent molecule and anti-tumor antibody. This kind of CAR-T cells, usually termed as “universal” anti-FITC CAR-T cells, bound FITC with specificity and was unable to recognize target cells. Thus, CAR-T cells were inactive unless the bispecific small molecules were added 47. Compared with traditional CAR-T cells, researcher reported that anti-FITC CAR-T cells induced by bispecific small molecules exhibited normal proliferation, cytotoxicity and anti-tumor activity with lower adverse effects. Using the approach of bispecific T cell engager, researchers can increase the safety of CAR-T cells treatment under control.

## Conclusions

Immunotherapy has demonstrated satisfactory clinical curative effect and is identified as desirable therapy method of cancer. In recent years, immunotherapies which include gene therapeutics, antibody therapy, adoptive cell therapy and other therapies were intensively researched and achieved dramatic breakthrough. Among adoptive cell therapies, genetically modified CAR-T cytotherapy emerged distinguished effect especially in hematopoietic malignancies. Recently, Tecartus (brexucabtagene autoleucel), as the third CAR-T cells drug, was approved by the FDA for the treatment of MCL. Despite of numerous encouraging results, CAR-T cell therapy were high expense, manufacture sophisticated and the prediction of its security still remains arduous.

Identification of robust biomarkers in corresponding hematopoietic malignancies is one of the strategies to improve therapeutic effect. B cell activating factor receptor (BAFF-R) demonstrated compelling preclinical results and showed cytotoxicity against multiple human lymphoma and leukemia cell lines, including CD19-negative variants. It is critical for combating B cell malignancies when CD19-targeted antigen of tumor cells is lost. Considering the cost and labor-intensiveness, studies began concentrating on allogeneic CAR-T infusion and some has ongoing clinical phase trials. The cytotoxicity after CAR-T cells infusion is always a challenge that impedes the further development. But there is one thing we should know that the modalities of CAR-T cells treatment are still in their early stages and definitely the extensive use of CAR-T cells therapy is faced with several issues in scientific knowledge before it is widely applied.

## Figures and Tables

**Table 1:** Main progresses of Car-T cell therapy

Time	Researcher/Product	Event	Institutec
1993	Zelig Eshhar	First-generation CARs (CD3ζ-based)	NCI, Rosenberg lab
2002	Michael Sadelain	Second-generation CARs (CD28-based)	Memorial Sloan Kettering Cancer Center (MSKCC)
2004	Dario Campana	Second-generation CARs (4-1BB-based)	National University of Singapore (NUS)
2010	Mcichel Sadelain	Third-generation CARs	Memorial Sloan Kettering Cancer Center (MSKCC)
2011	Carl June	First report of CD19 CAR therapy in CLL	University of Pennsylvania
2013	Carl June	First report of CD19 CAR therapy in ALL	University of Pennsylvania
2013	Hinrich Abken	Fourth-generation CARs (TRUCK)	University of Cologne
2017	Kymriah, Yescarta	FDA approval of CD19 CAR therapy	Novartis and Gilead (Kite)

**Table 2:** Car-T cell therapy for hematological malignancies

Disease	Full name	Antigen	Some Clinical trials
ALL	acute lymphoblastic leukemia	CD19/CD20/CD22/CD123	NCT04012879; NCT04049383; NCT04094766; NCT04016129
CLL	Chronic lymphoblastic leukemia	CD19	NCT04007029; NCT03960840
NHL	non-Hodgkin lymphoma	CD19/CD20	NCT03790891; NCT03497533; NCT04169932
ALCL	anaplastic large cell lymphoma	CD30	NCT03383965; NCT04008394
HL	Hodgkin lymphoma	CD19/CD30	NCT01087294; NCT04134325
MM	Multiple myeloma	CD269/CD138	NCT03672318; NCT04182581; NCT03271632
